# Cooperative signaling between Wnt1 and integrin-linked kinase induces accelerated breast tumor development

**DOI:** 10.1186/bcr2592

**Published:** 2010-06-21

**Authors:** Arusha Oloumi, Mykola Maidan, Frances E Lock, Howard Tearle, Steven McKinney, William J Muller, Samuel AJR Aparicio, Shoukat Dedhar

**Affiliations:** 1 Cancer Genetics and Developmental Biology, British Columbia Cancer Agency, 675 W. 10th Ave, Vancouver, BC V5Z 1L3, Canada; 2 Molecular Oncology and Breast Cancer Program, British Columbia Cancer Agency, 675 W. 10th Ave, Vancouver, BC V5Z 1L3, Canada; 3 Prostate Research and Jack Bell Research Centre, 2660 Oak St., Vancouver, BC V6 H 3Z6, Canada; 4 Department of Biochemistry and Molecular Biology, University of British Columbia, 6331 Crescent Rd., Vancouver, V6T 1Z3 BC, Canada; 5 Goodman Cancer Centre, McGill University, 1160 Ave Des Pins Ouest, Montreal, Quebec H3A 1A1, Canada

## Abstract

**Introduction:**

Breast cancer is genetically and clinically a heterogeneous disease. However, the exact contribution of different cell types and oncogenic mutations to this heterogeneity are not well understood. Recently, we discovered an interaction between Wnt and integrin-linked kinase (ILK) within the signaling cascade that regulates cell growth and survival. Interestingly, mammary-specific expression of either one of these proteins has been shown to promote mammary tumorigenesis. In light of our recent findings and to investigate the potential interaction between Wnt and ILK proteins during mammary tumor formation and progression, we established a transgenic mouse model that expresses both Wnt and ILK in mammary epithelial cells.

**Methods:**

A novel transgenic mouse model with mammary-specific expression of both Wnt1 and ILK was generated by crossing the two previously characterized mouse models, MMTV-Wnt1 and MMTV-ILK. The resulting MMTV-Wnt/ILK mice were closely monitored for tumor development and growth, as well as for the tumor onset. The molecular phenotypes of both tumors and premalignant mammary glands were investigated by using biochemical and global gene-expression analysis approaches.

**Results:**

A significant acceleration in mammary tumor incidence and growth was observed in the MMTV-Wnt/ILK mice. Pre-neoplastic mammary glands also display lobuloalveolar hyperplasia and an increase in ductal epithelium proliferation. Apart from elevated expression of Wnt/ILK targets, such as β-catenin and cyclin D1, gene-expression profiling identified the surprising activation of the FOXA1 transcription factor. Upregulation of FOXA1, which is also known as the molecular marker of differentiated mammary luminal cells, was consistent with the expansion of the enriched luminal progenitor population or CD29^lo^CD24^hi^CD61^+ ^cells in MMTV-Wnt/ILK tumors.

**Conclusions:**

These results show cooperation between *Wnt1 *and *ILK *transgenes during mammary carcinogenesis, leading to changes in a transcriptional network, which could dictate a specific breast cancer phenotype with enhanced growth dynamics. The MMTV-Wnt/ILK can be used as a model to identify further the genes downstream of the estrogen receptor-β/FOXA1 and to investigate the mechanisms targeting the expansion of the luminal progenitor cells leading to hyperplasia and tumorigenesis.

## Introduction

The oncogenic conversion of a primary epithelial cell to a malignant tumor involves cooperation between multiple signaling pathways, including the activation of growth-promoting signals and the inactivation of tumor-suppressor genes [1]. In breast cancer, a complex molecular interplay involving multiple signaling pathways, including Wnt signaling cascade [2], has been attributed to the development of this disease [3]. The Wnts comprise of a large family of secreted glycoproteins that affect diverse processes such as gene transcription, cell adhesion, and cell polarity [4]. The highly conserved canonic Wnt/β-catenin pathway activates β-catenin-dependent gene expression by regulating stabilization and nuclear accumulation of β-catenin, a protein originally identified as a component of intercellular junctions [5]. In the absence of the Wnt ligand, cytoplasmic β-catenin level is kept low through phosphorylation and ubiquitin-mediated proteosomal degradation by a multiprotein complex composed primarily of GSK-3β, Axin, and adenomatous polyposis coli (APC) (reviewed in [4,6]).

To date, evidence for mutations in the Wnt signaling-pathway components in breast cancer is lacking. However, various lines of evidence suggest that in breast cancer, Wnt signaling may be deregulated by loss of expression of negative-pathway regulators such as secreted Frizzled-related protein 1 (sFRP1), which is downregulated in many human breast tumors and is associated with poor prognosis [2,7]. Wnt-induced expansion of progenitor cells in mammary tumor development was recently identified [8-10]. In addition, stabilization of β-catenin and amplification of its target gene, *cyclin D1*, is documented in > 50% of breast carcinomas [11,12], suggesting that the downstream effectors of the Wnt cascade could also be activated through abnormalities in other signaling pathways. Integrin-linked kinase (ILK)-mediated signaling is one such pathway. ILK is a cytoplasmic effector of integrin receptors and is involved in oncogenesis through the induction of antiapoptotic pathways and cell-cycle progression [13,14]. Overexpression of constitutively active ILK in epithelial cells results in simultaneous inhibition of GSK-β activity and translocation of β-catenin to the nucleus, followed by an increase in β-catenin/Tcf transcriptional activity. Additionally, inhibition of ILK in colon carcinoma cells with constitutively active nuclear β-catenin resulted in inhibition of β-catenin/TCF transcriptional activity (reviewed in [15]). A similar correlation also was reported in human colon cancer between ILK expression, activation of β-catenin, and tumor progression *in vivo *[16]. In addition to the effect of ILK on the downstream components of the Wnt pathway in the absence of Wnt signaling, we demonstrated a significant reduction in Wnt-induced nuclear accumulation of β-catenin on ILK inactivation. Our results suggest a potential role of ILK in the regulation of Wnt-mediated β-catenin turnover and nuclear accumulation [17].

In light of these recent findings, and the fact that ILK activation and overexpression can promote oncogenic phenotypes in both cell-culture and transgenic mouse models [13], we have initiated *in vivo *experiments to determine whether ILK and Wnt signaling pathways interact to modulate tumor formation and progression. We have generated double transgenic mice that overexpress both ILK and Wnt1 in mammary glands. The MMTV-Wnt1 transgenic mice have been used extensively as a model of breast cancer development and progression [8,18,19]. Interestingly, MMTV-Wnt1-driven tumors show an enhancement in ILK expression and a requirement for ILK activity for cyclin D1 upregulation [20]. This suggests that overexpression of ILK may be required, not only for the initiation of premalignant changes, but also for the maintenance of the malignant phenotypes driven by Wnt signaling. MMTV-ILK transgenic mice also develop premalignant changes such as hyperplasia, as well as tumor formation, which occur with a much longer latency than in the MMTV-Wnt1 mice [21]. ILK overexpression has been reported in many human cancers, with a positive correlation with cancer growth and an inverse correlation with survival [22-24].

To test the *in vivo *functional significance of possible crosstalk between ILK and Wnt1 signaling pathways, we used a transgenic approach. Double-transgenic mice were generated that express both *Wnt1 *and *Ilk *transgenes in mammary epithelium under the mouse mammary tumor virus (MMTV)-long terminal repeat (LTR) promoter.

Here we report a significant acceleration in tumor onset, development, and growth rate in MMTV-Wnt/ILK double-transgenic mice as well as a profound enhancement in pre-neoplastic mammary gland hyperplasia. In addition, we provide unanticipated, novel evidence suggesting cooperation between the Wnt1 and ILK pathways, resulting in upregulation of FOXA1/ER-α transcriptional network and the expansion of the luminal progenitor cells, leading to accelerated breast cancer development.

## Materials and methods

### Generation of MMTV-Wnt/ILK transgenic mice

MMTV-Wnt1 (a gift from Dr. Harold Varmus) and MMTV-ILK [21] mice were backcrossed to the FVB strain. MMTV-Wnt1 males were then bred with MMTV-ILK female to generate the double-transgenic MMTV-Wnt/ILK mice. Mice were tail clipped and genotyped by PCR by using forward 5'-CCACACAGGCATAGAGTGTCTGC -3' and reverse 5'-GGACTTGCTTCTTATAGCC-3' primers for *Wnt1 *transgene [25] and forward 5'-CATGTATGCACCTGCCTG-3' and reverse 5'- TATGTCACACCACAGAAG 3' primers for the *ILK *transgene [21]. Nulliparous females were monitored biweekly for the appearance of a small palpable tumor nodule in the mammary fat pads. Ages were recorded at the time of tumor discovery and tumors were measured every 2 to 3 days with a calliper by using the modified ellipsoid formula *(LxW^2^)/2 *[26]. All animal procedures were done in accordance with protocols approved by the Institution Animal Care Committee at the Jack Bell Research Centre and University of British Columbia in Vancouver.

### Whole-mount mammary glands, histology, and immunohistochemistry

For whole-mount staining, the fourth mammary glands from transgenic and control mice were harvested and fixed in Carnoy's fixative (60% ethanol, 30% chloroform, 10% acetic acid) for 2 to 4 h, rehydrated and stained in carmine alum (Stem Cell Technologies, Vancouver, BC, Canada) overnight. After dehydration, samples were spread on a microscope slide, cleared in xylene, and images were acquired by using a dissecting microscope. For histologic analysis, mammary and tumor tissue samples were fixed in 10% neutral buffer formalin overnight at room temperature and were subsequently rinsed and kept in 70% ethanol until embedded in paraffin and sectioned at 5-μm thickness. Sections were either stained with hematoxylin and eosin (H&E) or processed for immunohistochemical analysis and counterstained by following standard procedures. Sections were incubated with rabbit anti FOXA1 (Abcam ab55178, Cambridge, MA), monoclonal β-catenin (BD-610153), polyclonal CD24 (BD-557436) (BD Transduction Laboratories, Mississauga, ON, USA), monoclonal PCNA (sc-56), polyclonal Ki-67 (Neomakers RM9106-S0) and polyclonal ER-α sc-542 (Santa Cruz Biotechnology Inc., Santa Cruz, CA) antibodies. Mouse and rabbit IgG antibodies were used as controls (Santa Cruz Biotechnology). All antibodies were used at 1:100 dilutions. Antibody binding was detected by using the Dako Cytomation Envision System (Dako Cytomation, Carpinteria, CA) and Vector NovaRED substrate kit (Vector Laboratories Inc., Burlingame, CA), according to the manufacturer's protocol.

### Preparation of tumor-cell suspension, cell sorting, and immunofluorescence

Tumors were finely chopped with a razor blade and then digested in DMEM containing 10% collagenase/hyaluronidase mixture (Stem Cell Technologies) for 1.5 h at 37°C. Antibodies used against mouse antigens were purchased from BD Pharmingen, unless otherwise specified, and included CD24-APC (BD-101813); CD-61-PE (BD-555735); CD29-FITC (Biolegend-12343); biotinylated CD31 (BD-553371), CD45 (Biolegend-103103), Ter119 (Biolegend-116203); and streptavidin-PE-Texas red (BD-551487). Antibody staining and cell sorting was performed as previously described [8]. For labeling of intracellular epitopes, cells were cultured on poly-L-lysine-coated coverslips, and were fixed in 4% paraformaldehyde and permeabilized in 0.2% Triton-X 100 for 4 min at room temperature before blocking in 2% BSA in PBS at room temperature for 30 min. Antibodies used were cytokeratin 14 (Covance, PRB155P) and cytokeratin 18 (Progen Biotechnik, GP CK18.2).

### Protein extraction and Western blot analysis

Mammary gland and tumor samples were flash frozen in liquid nitrogen and lysed in buffer containing 1% Triton X-100, 50 m*M *Hepes (pH = 7.5), 150 m*M *NaCl, 10% glycerol, and 1 m*M *and 2 m*M *EDTA supplemented with the appropriate protease and phosphatase inhibitor [27]. Protein concentrations were determined by using the BCA Protein Assay (Pierce, Rockford, IL, USA) according to the manufacturer's recommendations. Western blots were performed by using the following antibodies: monoclonal β-catenin (BD Transduction Laboratories 610153), monoclonal cyclin D1/D2 (Upstate Biotech 05-362, Lake Placid, NY), polyclonal Ruvb-like 1 (Protein Tech. Group 10210-2-AP, Chicago, IL), and monoclonal β-actin (Sigma A5441, St. Louis, MO) as loading control. All antibodies, unless otherwise indicated, were used at 1:1,000 dilution.

### Microarray

Gene-expression profiling was performed at the Centre for Translational and Applied Genetics (CTAG, Vancouver, BC, Canada) by using Affymetrix GeneChip Mouse Exon 1.0 ST Arrays according to the manufacturer's instructions. In brief, total RNA was extracted from the snap-frozen tumor tissue by using the GeneChip Whole Transcript (WT) Sense Target (ST) Labeling Assay (Affymetrix), as per manufacturer's recommendations, before being hybridized to a GeneChip Mouse Exon 1.0 ST Array (Affymetrix). RNA, 1 μg, was used as starting material, and the rRNA was reduced by using the RiboMinus Transcriptome Isolation Kit (Invitrogen), as per Affymetrix recommendations. Each chip was run through the GeneChip Fluidics Station 450 for washing and staining before scanning the chip on the GeneChip Scanner 3000 7G (Affymetrix). The corresponding microarray data were deposited into the Gene Expression Omnibus, with accession number GSE19300.

### Quantitative RT-PCR

Quantitative real-time PCR (Q-RT-PCR) was conducted in 384-well plates on an Applied Biosystems (Foster City, CA, USA) Q-RT-PCR instrument by using Roche Universal Probe Library (UPL) (Roche Applied Science, Laval, Quebec, Canada) according to the manufacturer's instructions. In brief, 1 μg of total RNA was used in a 40-μl reaction to make cDNA. Subsequently, 10 μl of the Q-RT-PCR mixture containing 100 n*M *UPL probe, 200 n*M *of each primer, and TaqMan PCR master mix (Applied Biosystems) was loaded into each well. After preliminary 95°C incubation, the samples were read for 40 cycles (95°C, 30 sec; 60°C, 30 sec; 72°C, 60 sec). The values for mRNA expression were normalized by using β-actin or GAPDH or both as the housekeeping genes. All Q-RT-PCR primers were designed by using the Roche Applied Science online assay design center for UPL system, and were purchased from Invitrogen (Burlington, ON, Canada). All assays were set up for a relative quantitation method in which mean Ct values from MMTV-Wnt1 samples were used as calibrators for data analysis for MMTV-Wnt/ILK samples. The relative fold change (FC) of gene expression between MMTV-Wnt1 and MMTV-Wnt/ILK samples was calculated by using the standard 2^-ΔΔct ^method.

### Statistical analysis

All the statistical analysis was performed as specified in this section, unless otherwise indicated in the figure legends. The percentage tumor-free data were plotted by using Kaplan-Meier curves, and differences in tumor free rates were assessed with the Cox model. Tumor-volume data were analyzed by using linear regression. Affymetrix GeneChip expression data were processed by using ArrayAssist software (ref ArrayAssist v1.x). Probe-set summarization and background correction of expression values was performed by using the RMA algorithm (ArrayAssist ExonRMA), and chip-to-chip variation was corrected by using quantile normalization. Quantitative RT-PCR data (cycle time or Ct data) were obtained by using RQ Manager Software (Applied Biosystems). Ct data were analyzed by using linear mixed effects, allowing for the biologic sample replicates and Q-RT-PCR plate-to-plate variation, as well as adjustment for to the reference loading control β-actin or GAPDH or both. Results were exponentiated to obtain natural scale fold-change estimates (that is, 2^-ΔΔCt^) and confidence intervals. The null-effect reference line at 1.0 is shown in the fold-change plots, and statistically significant fold changes are indicated by whisker 95% confidence intervals that do not cross the reference line. Benjamini-Hochberg (BH) adjustment of *P *values to correct for multiple comparisons was also performed on Q-RT-PCR data, and only genes with *P *value of < 0.05 were considered significantly differentially expressed.

## Results

### Mammary tumor formation and growth are accelerated in MMTV-Wnt/ILK double-transgenic mice

**Figure 1:**
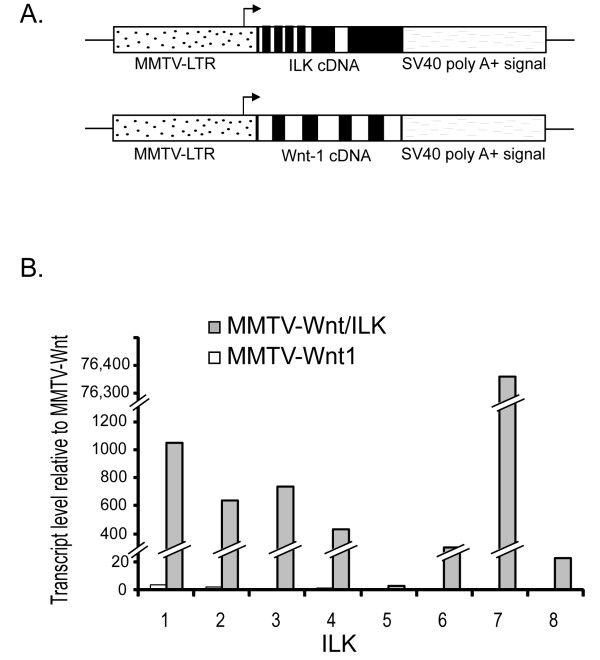
**Overexpression of the human ILK gene**. **(a**) Mouse MMTV-Wnt1 and human MMTV-ILK transgene constructs **(b)**. Quantitative RT-PCR performed on randomly selected tumors from each MMTV-Wnt1 and MMTV-Wnt/ILK group (*n* = 8) showing a dramatic overexpression of the human *ILK *gene in the double-transgenic samples. The bar graphs show the relative amount of human ILK overexpression in the individual MMTV-Wnt/ILK and MMTV-Wnt1 tumors normalized for β-actin expression. The statistical analysis with 95% confidence interval of (+754.18; -256.46) shows an average value of about 400-fold of overexpression in human ILK in MMTV-Wnt/ILK with an adjusted *P *value of 3.1E-16 determined by Benjamini-Hochberg test.

**Figure 2:**
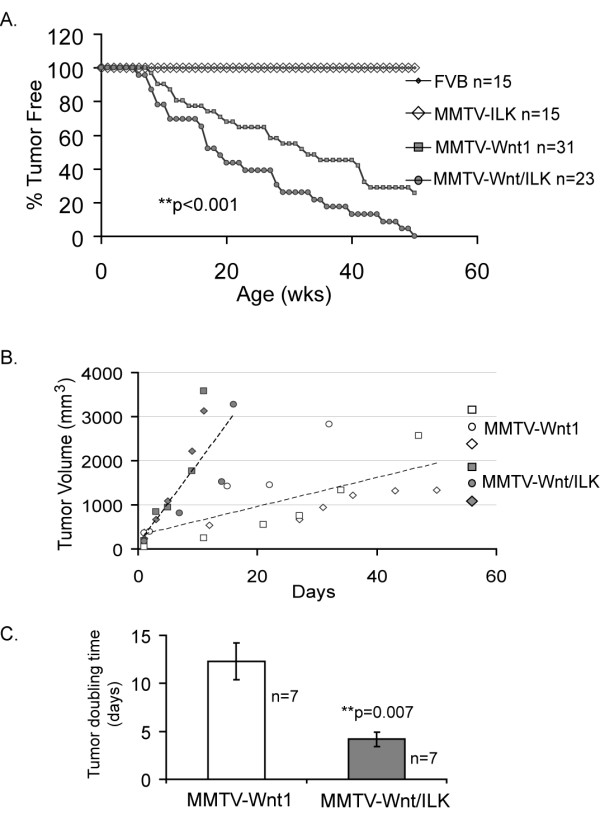
**Mammary tumor formation and tumor growth in MMTV-Wnt/ILK mice**. **(a) **Kaplan-Meier analysis of the percentage of tumor-free mice over time; *P *< 0.001. **(b) **Linear representation of growth rate from three randomly selected tumors from each transgenic group shows an accelerated rate of growth in MMTV-Wnt/ILK mice. **(c) **Tumor-doubling time was calculated based on the exponential curve fit of the tumor-volume measurements against time. The results show the mean doubling time for *n* = 7 tumors collected in each group, which supports the proliferative advantage of the tumors arising in the MMTV-Wnt/ILK transgenic group. The error bars represent the standard error of the mean (SEM), and the **P value was calculated by using a paired two-sample *t *test with a 95% confidence interval.

To begin investigating the cooperation between ILK and Wnt signaling pathways in mammary tumor formation we needed to determine how overexpression of ILK would affect Wnt1-induced mammary tumor development. Double-transgenic MMTV-Wnt/ILK mice were created by crossing the previously available MMTV-Wnt1 male and the MMTV-ILK female (see Materials and Methods) (Figure 1a). In addition to genotyping to confirm the presence of both transgenes (that is, MMTV-Wnt1 and MMTV-ILK) (Additional file 1, Figure S1), quantitative RT-PCR confirmed overexpression of human ILK in tumors of the double-transgenic mice (Figure 1b), whereas the level of Wnt1 overexpression remained fairly consistent in both transgenic models (data not shown). After genotyping, double-transgenic MMTV-Wnt/ILK female mice and their littermate controls (MMTV-Wnt1, MMTV-ILK, and FVB) were monitored and palpated weekly for the onset of tumors. The percentage of animals in each cohort remaining free of palpable mammary tumors was plotted at weekly intervals as a function of age for virgin females (Figure 2a). Mammary tumors appeared earlier in bi-transgenic animals overexpressing both *Wnt *and *Ilk *oncogenes. About half of the bi-transgenic mice examined (*n* = 23) developed palpable mammary tumors by the age of 19 weeks (T_1/2 _= 19), whereas fewer than 30% of the Wnt1 transgenic and none of the ILK or FVB control mice had tumors. Furthermore, by age 50 weeks, all of the bi-transgenic mice exhibited tumors, at which time about 25% of the Wnt1 transgenic mice (*n* = 31) still were tumor free (Figure 2a). In addition, the tumors that arose in both Wnt1 and Wnt/ILK transgenic mice were lobuloalveolar adenocarcinomas, as was previously reported for the Wnt1 transgenic mice.

To determine the growth rate after tumor onset, the sizes of the mammary tumors were periodically measured. We observed a significant difference in the growth rate of the mammary tumors between MMTV-Wnt1 and MMTV-Wnt/ILK transgenic mice. In general, tumors arising in MMTV-Wnt/ILK mice grew at an accelerated rate, forming large tumors of 2,000 mm^3 ^or more within 14 days, whereas tumors in MMTV-Wnt1 typically did not grow to this size in 50 days (Figure 2b). Therefore, based on the calliper measurement of individual tumors from each group, tumors from MMTV-Wnt/ILK mice showed a tumor-doubling time of 4 days, whereas the doubling time of the tumors in the MMTV-Wnt1 group was 3 times longer, averaging 12 days (Figure 2c).

### Pre-neoplastic mammary glands of MMTV-Wnt/ILK mice show enhanced hyperplasia and proliferation

**Figure 3:**
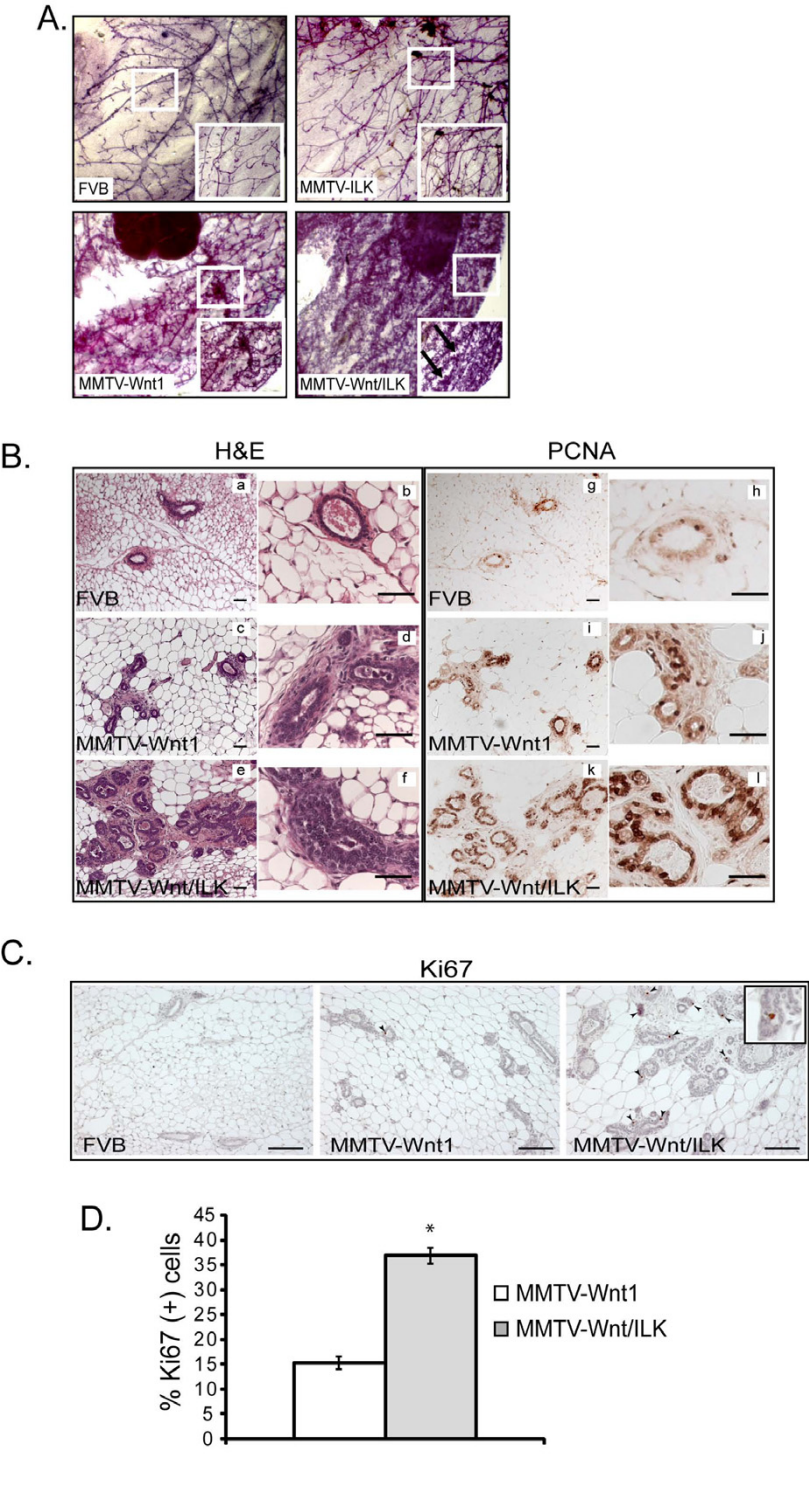
**Pre-neoplastic mammary gland analysis**. **(a**) Whole-mount ductal outgrowths from 10- to 12-week-old FVB, MMTV-Wnt1, and MMTV-ILK mice and 8-week-old MMTV-Wnt/ILK mice show a marked enhancement in the ductal network of MMTV-Wnt/ILK pre-neoplastic mammary glands at an earlier age. Images were taken by using a dissecting microscope at 4× magnification. Black arrow shows an area of dense alveoli. **(b) **Sections from inguinal mammary glands of 8- to 9-week-old mice were stained with H&E and PCNA. Mammary gland taken from an FVB mouse shows a normal glandular epithelial **(a,b) **and minimal PCNA staining **(g-h)**, whereas sections from MMTV-Wnt show more hyperplasia **(c,d) **and more PCNA staining **(i,j)**. Section of mammary glands from MMTV-Wnt/ILK mice shows an even greater epithelial hyperplasia, multilayered and disorganized ductal epithelium **(e,f)**, as well as a marked increase in PCNA staining **(k,l)**. The pictures are representative of at least five different stainings. Scale bars, 50 μm. **(c) **Ki67 was used as an additional marker to confirm the higher proliferation status of the MMTV-Wnt/ILK pre-neoplastic mammary glands. Scale bars, 100 μm. **(d) **Ki67-positive staining is quantified based on the number of the mammary lumina containing Ki67-positive cells. At least 50 mammary lumina were counted in each condition in randomly selected fields of view. Data represent mean ± SD. *P < 0.04 was calculated by using a paired two-sample *t* test.

To determine whether the observed accelerated tumor incidence starts with a higher proliferation during mammary gland development, we examined the pre-neoplastic mammary glands in all transgenic models. The mammary gland architecture was therefore analyzed with whole-mount preparation. Whereas the mammary glands taken from a wild-type FVB female mouse revealed a branching ductal structure typical of a virgin female, reflected by the presence of an ordered ductal network with side branching, the pre-neoplastic mammary glands from MMTV-Wnt/ILK transgenic mice exhibited increased ductal branching with more secondary and ternary side branches, as well as a denser alveolar development at an earlier age (8 weeks old) compared with either MMTV-Wnt1 mice or controls (10 to 12 weeks old) (Figure 3a). The areas of dense alveoli are indicative of mammary gland hyperplasia (black arrows in Figure 3a). In addition, the presence of hyperplastic mammary tissue was confirmed by hematoxylin and eosin (H&E) staining of transgenic mammary gland sections (Figure 3b, a-f). The mammary glands of MMTV-Wnt/ILK mice showed a marked enhancement in epithelial hyperplasia as well as multilayered and disorganized ductal epithelium, compared with the mammary glands of the MMTV-Wnt1 mice.

Immunohistochemical staining also was used to examine the pre-neoplastic mammary glands for both PCNA (proliferating cell nuclear antigen)- and Ki67-positive cells, two factors known to represent proliferation. Whereas PCNA is maximally detected in the S phase, Ki67 is preferentially expressed during later stages of the cell cycle [28], providing different staining patterns, but both indicative of the proliferation status of the tissue. As previously shown [9], we also confirmed a greater proliferation status in the mammary gland cells in MMTV-Wnt1 transgenic mice compared with non-transgenic FVB controls (Figure 3b, g-j). However, the mammary glands of MMTV-Wnt/ILK mice showed an even greater increase in both PCNA (Figure 3b, g-j) and Ki67 (Figure 3c) staining over those observed in the glands of MMTV-Wnt1 transgenic mice (Figure 3b, i-l, and 3c). Altogether, these results suggest a further enhancement in the proportion of the proliferating cells as the result of the mutual input from both ILK and Wnt signaling pathways in the double-transgenic mice. The pre-neoplastic mammary glands of the double-transgenic mice on average showed about a twofold increase in proliferation activity based on Ki67 staining compared with the glands of the MMTV-Wnt1 model (Figure 3d).

### Biochemical analysis of the double-transgenic tumors reveals upregulation of proliferation promoting molecular markers

**Figure 4:**
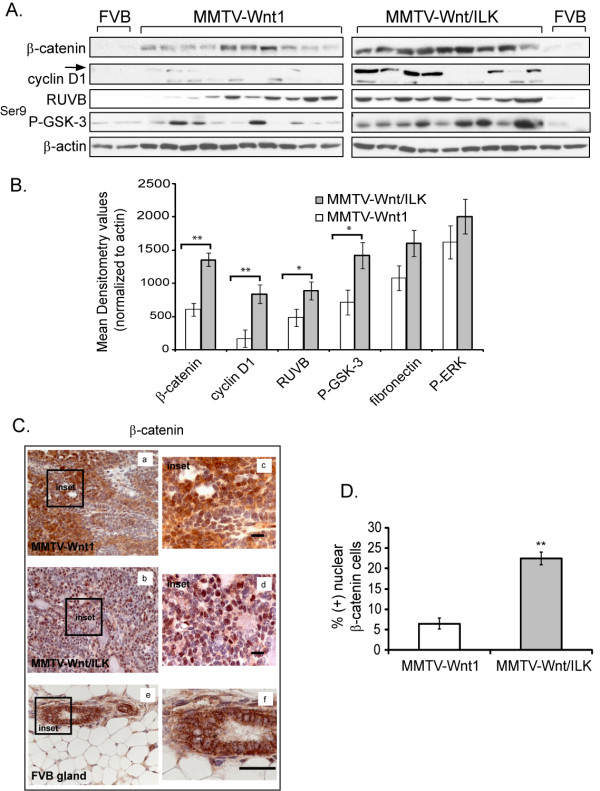
**Biochemical analysis, densitometry results, and immunochemistry**. **(a) **The biochemical analysis of the tumors from each group demonstrates upregulation of certain molecular markers in MMTV-Wnt/ILK tumors, which could potentially lead to the more-aggressive growth phenotype observed in these tumors. The lysates from two different FVB mammary glands were used as controls. The blots were generated at the same time, by using the same apparatus, and under identical exposure times and conditions to ensure comparability. **(b) **The graph for the Western-blot analysis illustrates the densitometry results of the blots. Results represent the mean of densitometry data from 10 independent tumors normalized for β-actin expression. The error bars represent the standard error of the mean (SEM). *P ≤ 0.05; **P < 0.005; calculated by using an ANCOVA analysis. **(c) **β-catenin immunohistochemistry shows a significant increase in nuclear localization in excised tumors from MMTV-Wnt/ILK mice, suggesting a more-active β-catenin-mediated transcriptional machinery. Magnification: **(a,b) **40×; **(c,d) **63×; *n* = 4. β-catenin staining of the normal FVB mammary gland is included as a control **(e,f)**. Scale bars, 25 μm. **(d) **Positive nuclear β-catenin staining was determined by counting the cells that showed overlapping hematoxylin and β-catenin staining. The cells were counted in five equal randomly chosen fields of view at 40× magnification in four individual tumors. Results are reported as the mean of the ratio of the total number of the nuclear-positive cells to the total cell count. The error bars represent the standard error of the mean (SEM). **P < 1.1 × 10^-7 ^was calculated by using a paired two-sample *t *test.

To investigate the potential role of some of the known downstream targets of both Wnt and ILK signaling pathways in the enhanced development of tumors in Wnt/ILK double-transgenic mice, we performed a series of Western blots on protein extracts isolated from 20 randomly chosen tumors. In addition, we included protein extracts from two normal mammary glands harvested from FVB mice as a control (Figure 4a). The Western-blot data were quantified by densitometry analysis (Figure 4b). All values were normalized to β-actin for the corresponding sample on each gel. We observed a clear difference in the expression of some of the downstream molecular effectors of the Wnt signaling pathway, such as cyclin D1, β-catenin, and suggestive evidence of a difference for RuvB-like 1. Western-blot analysis of β-catenin showed greater than twofold increase in β-catenin expression in Wnt/ILK double-transgenic tumors compared with Wnt1-induced mammary tumors (Figure 4a,b). More interesting, β-catenin immunohistochemical staining revealed a marked increase in nuclear accumulation of β-catenin in MMTV-Wnt/ILK tumors compared with a more-cytoplasmic distribution of β-catenin observed in Wnt1 tumors (Figure 4c). It is known that an increase in Wnt-mediated β-catenin is attributable to increased stability through the inhibition of the ubiquitin-mediated degradation [29,30]. In MMTV-Wnt/ILK tumors, further activation of ILK, which has also been shown to regulate β-catenin stabilization and nuclear localization (reviewed in [15]), further contributed to this increase. Quantification of the proportion of the cells staining positive for nuclear β-catenin to the total number of the cells in five randomly chosen fields of view shows a 3.5-fold increase in nuclear positive β-catenin staining in MMTV-Wnt/ILK tumors (Figure 4d). In addition, RuvB-like 1, a TATA box-binding protein essential for cell growth [31], which participates in β-catenin-mediated transactivation by binding to nuclear β-catenin [32], also showed about a 1.8-fold increase in MMTV-Wnt/ILK tumors. Interestingly, RuvB-like 1 was also recently found to interact with ILK in a proteomics analysis for the identification of the components of the ILK interactome [33].

Previous studies have shown an increased abundance of cyclin D1 in Wnt1 mammary tumors [34,35]. Although cyclin D1 levels were increased in Wnt1 tumors compared with normal mammary epithelium, we found an additional fourfold increase in the cyclin D1 level in Wnt/ILK double-transgenic mammary tumors. We also observed an increase in GSK-3 Ser-9 phosphorylation in MMTV-Wnt/ILK tumors. Both cyclin D1 and phosphorylation of GSK-3β are regulated by ILK as well as by Wnt1 [20]. Other signaling molecules, such as P-ERK and fibronectin, also showed a marginal increase in the MMTV-Wnt/ILK tumor samples (Figure 4b). However, this upregulation was not statistically significant with the sample size tested.

Some of the other downstream targets of Wnt and ILK pathways, such as c-myc, Lef-1, MMP-2, MMP-7 (data not shown), ^ser473^P-AKT (Additional file 1, Figure S2), did not show a marked difference in expression in the mammary tumors between the two transgenic groups. Overall, these data suggest activation of collaborative oncogenic signaling pathways contributing to the more-aggressive growth phenotype of the MMTV-Wnt/ILK tumors.

### Transcriptional profiling of transgenic mammary tumors

To identify other possible signaling molecules and events involved in increased tumor incidence in MMTV-Wnt/ILK transgenic tumors, we performed global gene-expression analysis on RNA isolated from four independent tumors from each MMTV-Wnt1 and MMTV-Wnt/ILK transgenic model. Gene-expression levels were determined by using the Affymatrix GeneChip Mouse Exon 1.0 ST. The exploratory expression data obtained from the Affymetrix scanner were processed by using ArrayAssist software (ArrayAssist v1.x) controlling for false-negative and false-positive results. Probe-set summary and background correction of expression values were performed by using the RMA algorithm (ArrayAssist ExonRMA), and chip-to-chip variation was corrected by using quantile normalization. From the gene-chip data, we identified 200 known or predicted genes showing a significant fold change (data not shown). Volcano or scatterplot was used to highlight the most differentially expressed transcripts as the function of significance (determined based on *P *value) over differential expression (determined as fold change) (Additional file 1, Figure S3). Subsequently, all exon-level and gene-level probe sets showing an absolute fold change > 2.5 and an unadjusted *P *value < 0.05 were reviewed (Table S1). Categorization of these differentially expressed genes according to their molecular-function groups, as shown in Additional file 2, suggests a change in the program of gene expression, and particularly a transcriptionally active signaling network in Wnt/ILK mammary tumors (Additional file 2). Included among these are Sirt1, Hey2, Ncoa4, and Foxa1, all of which have been implicated in the progression of luminal breast cancer and ER-α-dependent signaling, as well as ER-α (that is, Esr 1) (Additional file 2). Ncoa4 (nuclear receptor co-activator 4) and FOXA1 (forkhead-box A1), both have been shown to act as transcriptional activators of estrogen (ER)-α [36,37]. Sirt1 transcription factor has been shown to regulate Hey2, a downstream effecter of Notch1 signaling [38,39], one of the more prominent activated pathways in the luminal-restricted mammary progenitor cells [40,41]. This functional classification also facilitated the selection of the gene candidates for further validation (Additional file 2). This microarray analysis with the limited number of chips used was an initial approach in obtaining an exploratory list of potential gene targets. We, therefore, followed up by selecting 26 of these genes for validation by quantitative reverse transcriptase PCR (Q-RT-PCR).

**Figure 5:**
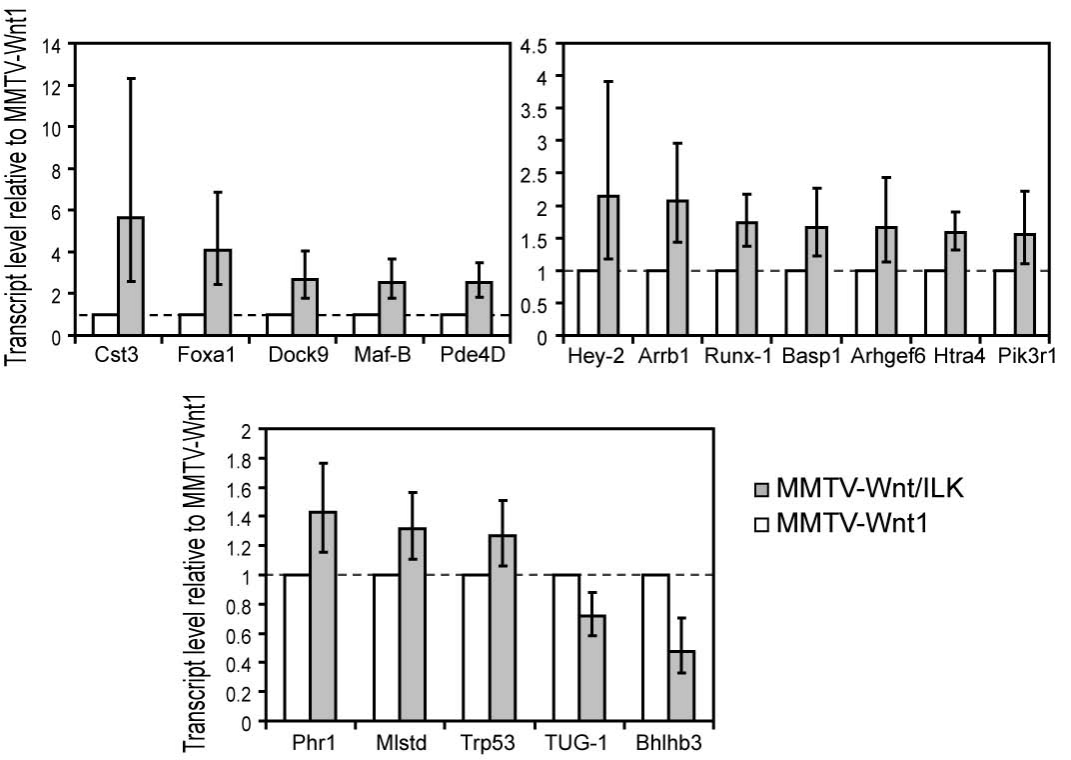
**Q-RT-PCR validation of the microarray data shows differential expression of 17 of 26 genes tested (at least three and up to eight samples)**. Results were exponentiated to obtain natural-scale fold-change estimates (that is, 2^-ΔΔCt^) and 95% confidence intervals, shown as bar heights and whiskers, respectively. The genes that showed a fold change of ≥ 30% with *P *≤ 0.05 are presented as validated genes.

Transcript levels for each test gene were normalized to β-actin or GAPDH transcript levels or both in the same extract. Logarithmic results were exponentiated to obtain linear-scale fold-change estimates (that is, 2^-ΔΔCt^) and confidence intervals. The results of the Q-RT-PCR assays demonstrated consistent differential expression for 17 of the 26 genes identified and tested from the Affymetrix analysis (Figure 5).

### MMTV-Wnt/ILK transgenic mice develop tumors enriched for luminal progenitor cells and active FOXA1/ER-α network

**Figure 6:**
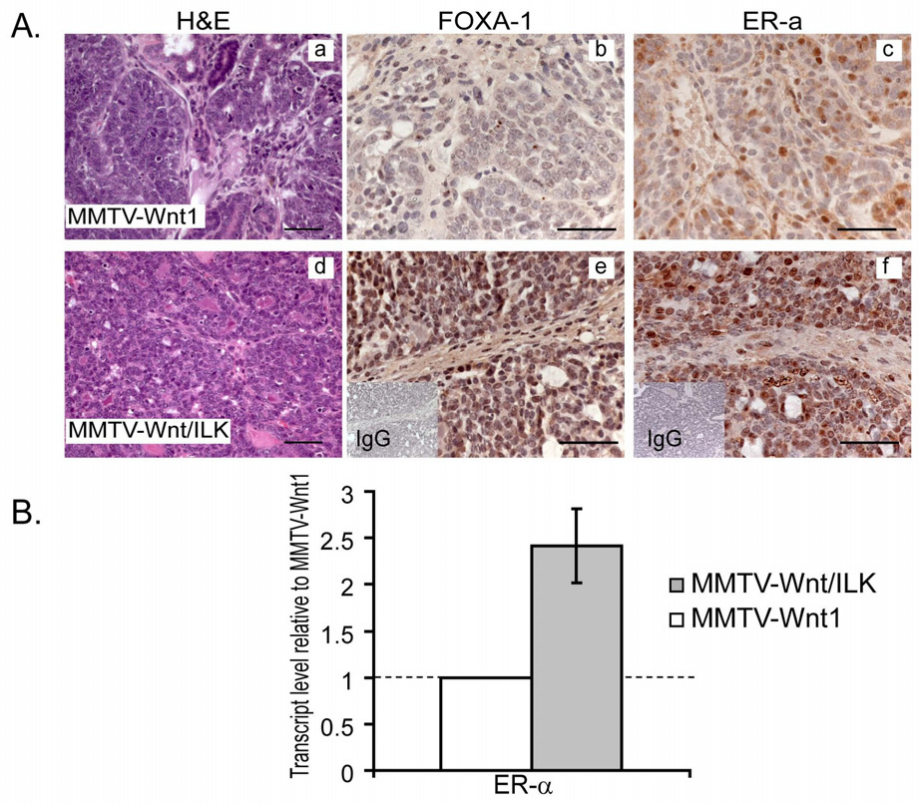
**Immunohistochemical staining and PCR analysis**. **(a) **Immunohistochemical staining of tumors from MMTV-Wnt/ILK mice exhibit strikingly enhanced expression of FOXA1 and ER-α. Magnifications: (**a,d) **40×; (**b,c, and e,f) **63×. Scale bars, 50 μm. **(b) **Q-RT-PCR analysis shows a 2.4-fold increase in ER-α transcripts levels in MMTV-Wnt/ILK tumors. Results are shown as fold change (FC) of ER-α expression in MMTV-Wnt/ILK over MMTV-Wnt1 tumors.

Expression profiling, by microarray DNA analysis, revealed a marked upregulation in FOXA1 in mammary carcinomas of the Wnt/ILK double-transgenic mice (Table S1). Upregulation of FOXA1 also was confirmed by Q-RT-PCR in a larger tumor sample set (*n* = 8) as well as at the protein level by immunohistochemical staining (Figure 6). Quantitative RT-PCR showed an average increase of fourfold in FOXA1 expression (Figure 5). The fact that FOXA1 controls the downstream transcription of ER-regulated genes [37,42,43] led us to examine further the expression of ER-α, which was also identified in the Affymetrix analysis. ER-α also showed evidence of fold change in our exon-level analysis on eight different exon probe sets, with an average fold change of 2.56 and a *P *value of 0.031.

At the protein level, although the tumors from both transgenic models were ER-α positive by immunohistochemistry, a higher level of staining was evident in sections from the double-transgenic tumors (Figure 6a). In addition, Q-RT-PCR revealed a 2.4-fold increase in ER-α expression at the mRNA level in MMTV-Wnt/ILK tumors (Figure 6b). Because the expression of ER-α is considered one of the hallmarks of luminal-type breast tumor [42], and FOXA1 is considered a marker of luminal cell differentiation during normal mammary gland development [44], we sought to examine the potential difference in the level of mammary gland luminal epithelial fraction in the two transgenic models.

Recently, CD24 was used as a single marker for isolation of pure populations of the luminal and basal compartments of the mouse mammary epithelium. Cells with a high expression of CD24 were identified to represent the mouse mammary luminal epithelial cells [41,45,46]. Other studies showed high expression of CD24 on luminal-restricted colony-forming cells [41,45,46]. In our model, initial immunohistochemistry revealed a greater presence of CD24-positive cells in both mammary tumors and pre-neoplastic mammary glands of MMTV-Wnt/ILK mice compared with the same tissue type in MMTV-Wnt mice (data not shown). This suggests a possible role for ILK in the background of MMTV-Wnt1 in expanding the luminal cell lineage with markedly enriched tumor-forming capacity.

**Figure 7:**
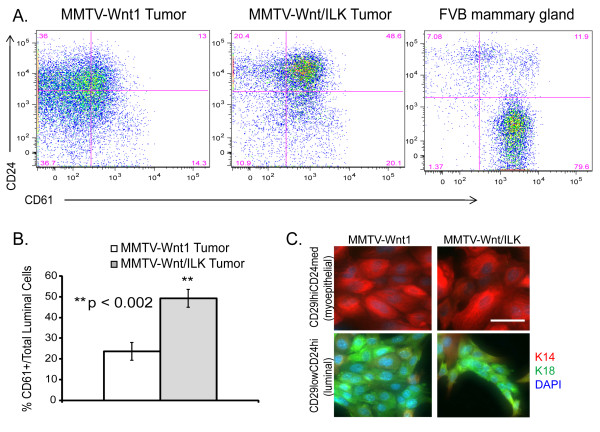
**Flow-cytometry analysis and relative proportions of cell types**. **(a**) Flow-cytometry analysis showing the distribution of Lin^-^CD29^lo ^for the expression of the surface markers CD24 and CD61. Mammary glands from 10-week-old FVB mice were included as a control for the expression of these cell-surface markers and analysis by FACS. The increase in CD24^hi^CD61^+ ^luminal progenitor cells is evident in the tumor cells of the MMTV-Wnt/ILK transgenic model compared with MMTV-Wnt (*n* = 3 per group). **(b) **Bar chart depicting the relative proportion of the CD61^+ ^luminal progenitor cells to the total luminal population (CD29^lo^CD24^hi^) in the two tumor types. Error bars indicate mean ± SD. **P < 0.002 was calculated by using a paired two-sample t test. **(c) **The purity of the cell populations (myoepithelial and luminal) in the sorted fractions is shown by the lineage-specific markers, K18 and K14, in the cultured tumor cells of both transgenic models. Scale bars, 25 μm.

However, because analysis of CD24 level by IHC is not an optimal way for such a comparison to be made, we further examined different epithelial subpopulations in tumors from both transgenic models with FACS analysis. We specifically looked at the expression of the cell-surface markers CD24 and CD61 within the Lin^-^CD29^lo ^fraction (Figure 7a). Within the CD29^lo^CD24^hi ^fraction, also known as the luminal subpopulation [47], we determined the level of CD61^+^, which is the enriched fraction for the luminal progenitor cells [8,48] (Figure 7a). Single-cell suspensions from mammary glands of FVB mice were used as a control for staining with these cell-surface markers (Figure 7a). As suspected, single-cell suspensions prepared from MMTV-Wnt/ILK tumors (*n* = 3), depleted of hematopoietic and endothelial cells [8] (referred to as "Lin^-"^), showed a more than two fold expansion in the percentage of CD61^+ ^luminal progenitor cells within the total luminal fraction, CD29^lo^CD24^hi ^population (Figure 7a,b). The purity of the cell populations was evaluated by determining the expression of the luminal lineage marker keratin 18 (K18) and the myoepithelial marker keratin 14 (K14) (Figure 7c). As expected, K18 expression was highest in the CD29^lo^CD24^hi ^luminal fraction, whereas expression of K14 was restricted to CD29^hi^CD24^med ^myoepithelial fraction in both transgenic models (Figure 7c). These data further suggest the expansion of the luminal progenitor lineage as the result of the mutual input of ILK and Wnt1 signaling.

## Discussion

Transgenic and knockout mice bearing genetic changes have been used as the conventional approaches to test some of the signaling events involved in the induction and progression of breast cancer. ILK- and Wnt-mediated signaling are two such pathways. Cooperative signaling between ILK and Wnt in cell culture was identified by us earlier [17,49]. In this study, we showed that mammary-specific expression of ILK in MMTV-Wnt1 mice results in the accelerated development of the spontaneous mammary tumors. Enhanced expression of pro-proliferative genes, such as *β-catenin *and *cyclin D1*, are evident in these double-transgenic mice. We also present several lines of evidence supporting the notion that the cooperative signaling events between Wnt1 and ILK pathways result in a highly active ER-α signaling pathway. The molecular signature of MMTV-Wnt/ILK tumors contained the transcripts of several proteins involved in ER-α signaling. Among those is FOXA1, which shows an average fourfold increase in expression in double-transgenic mammary tumors compared with Wnt1 tumors with > 95% confidence interval. Even though the transcriptional regulation of FOXA1 remains a subject of further investigation, earlier studies show positive regulation of FOXA1 by activated β-catenin through Sox17 [50]. Therefore, the Wnt1/ILK-mediated upregulation of nuclear β-catenin shown both *in vitro *[17] and *in vivo *(Figure 4) could potentially be responsible for the observed upregulation of FOXA1 expression in the Wnt1/ILK mammary tumors.

Forkhead-box A1 (FOXA1), a member of the "winged helix" family of the transcription factors, can regulate many genes associated with regulation of cell signaling and the cell cycle [51] and can act either as a growth stimulator or as a repressor. As a stimulator, FOXA1 interacts with *cis*-regulatory regions in heterochromatin and opens the chromatin [52], which subsequently enhances the interaction of ER-α with promoters of various ERα-regulated genes. Transactivations of ER-α machinery, as well as the enhancement in nuclear accumulation of β-Catenin also support the observed upregulation of cyclin D1 in Wnt1/ILK double-transgenic mammary tumors. *Cyclin D1 *is one of the most commonly overexpressed genes in human breast cancer whose transcription is activated by FOXA-1-mediated ER-α gene regulation [53,54], as well as by β-catenin-mediated transcriptional machinery [55]. Although the enhancement in cyclin D1 upregulation is not apparent at the transcript level with our microarray analysis, it is noteworthy that this approach was applied as the initial exploratory finding, and the small sample size used is not necessarily the best representative of some of the subsequent differences explored in this study.

Our gene-expression profiling also shows a significant decrease in multiple androgen receptor (AR) transcripts in MMTV-Wnt/ILK tumors (Additional file 3), which also supports the notion of more-active ER-α signaling machinery. Decreased AR signaling has been shown to correlate with an increase in ER-α transactivation as the result of shared co-activator proteins [56]. One such co-activator is identified as ARA70 or NcoA4 [36], another one of the genes identified in our expression analysis to be upregulated in MMTV-Wnt/ILK tumors. Overall, these results suggest a potential role for activated ER-mediated signaling in the biology of these fast-growing double-transgenic tumors.

Although hierarchic cluster analysis of breast-tumor subtypes with distinct gene-expression patterns has associated ER-α positive and FOXA1 expression with luminal subtypes [57], recently, aberrant luminal progenitors, positive for ER-α expression, were identified as the target of transformation for certain human basal tumors [58]. In our model, FACS profile analysis of tumor cells from the double-transgenic mice also clearly shows an expansion in the CD29^lo^CD24^hi^CD61^+ ^luminal progenitor cells. These results, along with a marked increase in CD24 expression in MMTV-Wnt/ILK tumors, provides evidence for tumors arising from the luminal lineage. Whereas CD24 expression has been implicated in multiple cell properties of direct relevance to tumor growth, such as tumor-cell proliferation [59], its high expression has been reported on luminal-restricted mouse mammary progenitor cells [48,60] and, more recently, on human luminal-restricted progeny through cluster analysis of gene-expression profiling [41]. Interestingly, some of our other data, such as enhancement of the downstream effectors of Notch1 signaling, Hey2 and Sirt1, further support the expansion of the population of the cells from the luminal lineage in MMTV-Wnt/ILK mammary glands and tumors. Notch1 and its target genes were recently shown to be preferentially active in both mouse and human mammary luminal progenitor cells *in vivo *[41]. Notably, ILK activity has been identified as a prosurvival event upstream of Notch1 signaling in a leukemic microenvironment [61]. Therefore, the cooperative events between Wnt and ILK signaling are likely to target the luminal progenitor cells for expansion and self-renewal as the initial steps during transformation. This provides a likely explanation for a mechanism involved in accelerated tumor development and growth identified in this model.

These results, together with the molecular-profiling analyses presented in this work, implicate luminal progenitor cells as the probable target population in MMTV-Wnt/ILK mammary tumors with extensive proliferation capacity.

Last, about 70% of human breast cancers are phenotyped as ER positive and are classified as the "luminal epithelial-like/ER-positive" subtype, which express high levels of ER-α and genes regulated by estrogen. However, it is clear that ER-positive tumors do not represent a single entity [58,62]. Therefore, identification of distinct progenitor cell populations with different hormonal sensitivities in the backbone of certain signaling events, such as cyclin D1, is an important step in determining the cellular origin of certain types of breast cancer and the successful development of targeted therapies.

## Conclusions

The exact contribution of different cell types and oncogenic mutations to breast cancer heterogeneity remains unknown. Here, we report novel crosstalk between ILK and Wnt1 oncogenes, resulting in accelerated breast cancer development with molecular characteristics consistent with a more-aggressive phenotype *in vivo*. The Wnt1/ILK transgenic model of mammary tumor development shows evidence of expansion of the luminal progenitor cells with a highly active ER-α transcriptional network. This could serve as model to study ER-α/FOXA1-mediated gene regulation, and to refine further the molecular classification of ER(+)-type breast cancers.

## Abbreviations

ER: estrogen receptor; FC: fold change; ILK: integrin-linked kinase; MMTV: mouse mammary tumor virus; Q-RT: quantitative reverse transcriptase; UPL: universal probe library.

## Competing interests

The authors declare that they have no competing interests.

## Authors' contributions

AO participated in the design of the study and acquisition of data, directed the data analysis, and drafted the manuscript. MM, FL, and HT participated in the acquisition of data and coordinated the data collection. SM performed statistical analyses and participated in interpreting results. WJM and SAJRA participated in the development of the study methods and coordinated the data collection. SD participated in the study design, co-directed the data analysis, and participated in interpreting results. All authors read and approved the final manuscript.

## Supplementary Material

Additional file 1 **Figures S1 to S3**. (S1) PCR amplification from the tail clipped DNA shows the appropriate size bands for both transgenes of interest (MMTV-Wnt1 and MMTV-ILK) in positive double-transgenic mice. (S2) Western-blot analysis of ^ser473^P-AKT did not show a marked difference in the expression in the mammary tumors between the two transgenic groups (S3) Volcano plot representing the microarray data or scatterplot of gene-expression data from Affymetrix analysis shows significance (*P *value) versus fold change of MMTV-Wnt/ILK to MMTV-Wnt1 gene expression tumor data in four individual tumors per group. The yellow boxes show the genes for which the up or down fold change was greater than 2, with a *P *value ≤ 0.01. Click here for file

Additional file 2 **Table S1**. Functional classification of the highly differentially expressed genes identified through Affymetrix transcript analysis of four tumors in each MMTV-Wnt/ILK and MMTV-Wnt1 transgenic mouse. Results show fold change (FC) ≥ 2.5 with a *P *value of < 0.05 of MMTV-Wnt/ILK tumors versus MMTV-Wnt1. Click here for file

Additional file 3 **Table S2**. Androgen-regulated transcripts identified in Affymetrix gene-expression analysis to be downregulated in MMTV-Wnt/ILK tumor samples. Results show fold change (FC) decrease of gene expression in MMTV-Wnt/ILK tumors versus MMTV-Wnt1. Click here for file
