# Supplementary figures and images for: Cooperative signaling between Wnt1 and integrin-linked kinase induces accelerated breast tumor development
Source: Breast Cancer Res. 2010 Jun 21;12(3):R38. doi: 10.1186/bcr2592 (PMC2917033; doi:10.1186/bcr2592)

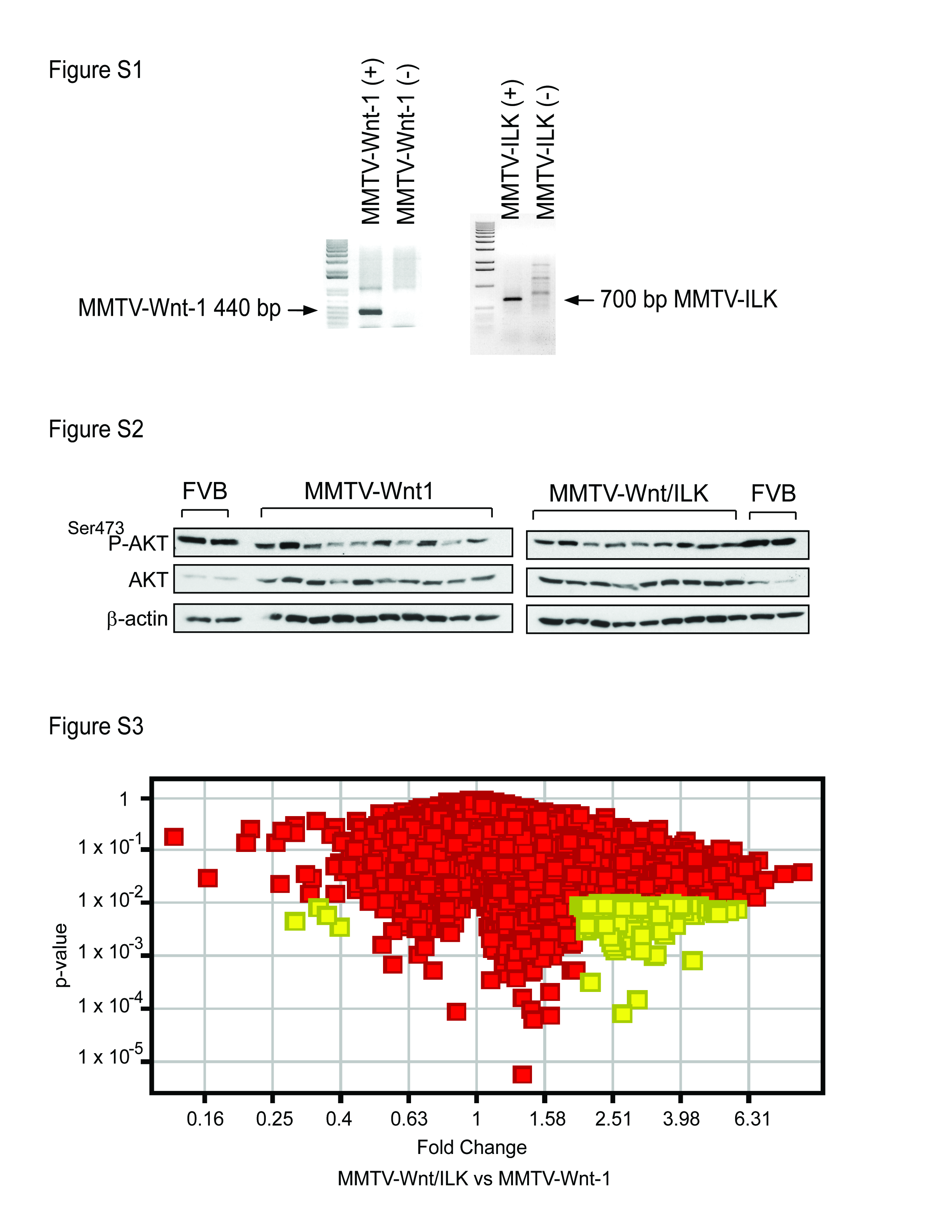

Supplement: Additional file 1 — Figures S1 to S3. (S1) PCR amplification from the tail clipped DNA shows the appropriate size bands for both transgenes of interest (MMTV-Wnt1 and MMTV-ILK) in positive double-transgenic mice. (S2) Western-blot analysis of ser473P-AKT did not show a marked difference in the expression in the mammary tumors between the two transgenic groups (S3) Volcano plot representing the microarray data or scatterplot of gene-expression data from Affymetrix analysis shows significance (P value) versus fold change of MMTV-Wnt/ILK to MMTV-Wnt1 gene expression tumor data in four individual tumors per group. The yellow boxes show the genes for which the up or down fold change was greater than 2, with a P value ≤ 0.01. [file bcr2592-S1.TIFF]

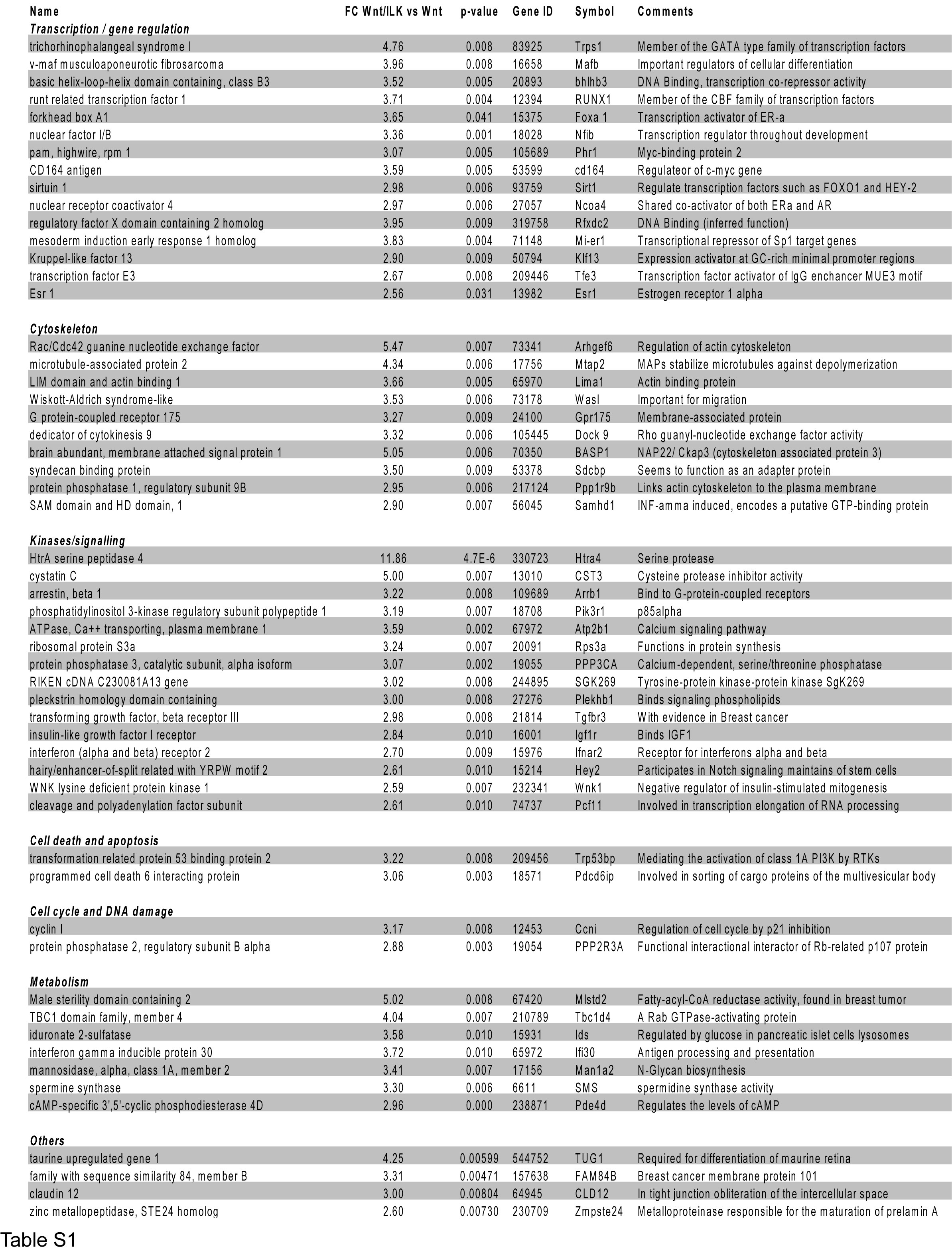

Supplement: Additional file 2 — Table S1. Functional classification of the highly differentially expressed genes identified through Affymetrix transcript analysis of four tumors in each MMTV-Wnt/ILK and MMTV-Wnt1 transgenic mouse. Results show fold change (FC) ≥ 2.5 with a P value of < 0.05 of MMTV-Wnt/ILK tumors versus MMTV-Wnt1. [file bcr2592-S2.JPEG]

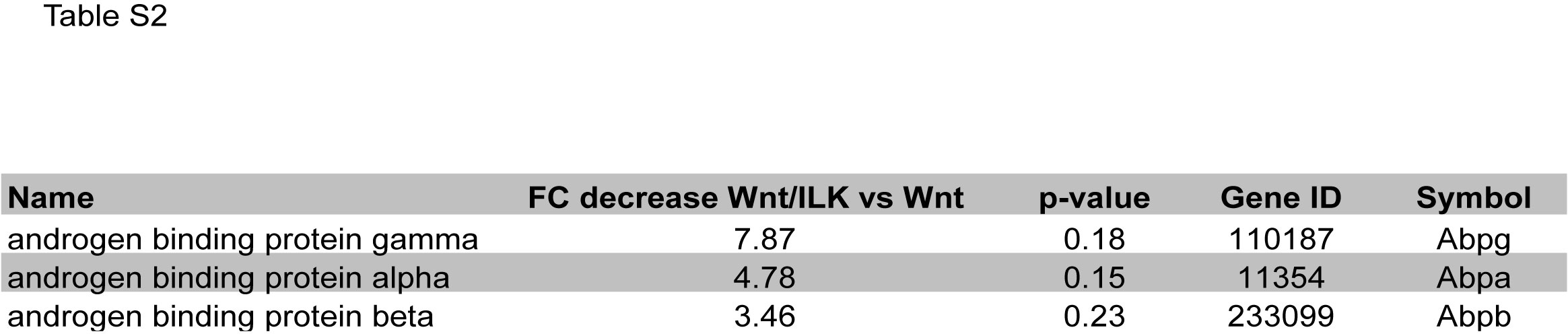

Supplement: Additional file 3 — Table S2. Androgen-regulated transcripts identified in Affymetrix gene-expression analysis to be downregulated in MMTV-Wnt/ILK tumor samples. Results show fold change (FC) decrease of gene expression in MMTV-Wnt/ILK tumors versus MMTV-Wnt1. [file bcr2592-S3.JPEG]
